# Deformation Behavior and Properties of 7075 Aluminum Alloy under Electromagnetic Hot Forming

**DOI:** 10.3390/ma14174954

**Published:** 2021-08-30

**Authors:** Zhihao Du, Zanshi Deng, Xiaohui Cui, Ang Xiao

**Affiliations:** 1 College of Mechanical and Electrical Engineering, Central South University, Changsha 410083, China; dzh7695782@126.com (Z.D.); dzscsu@csu.edu.cn (Z.D.); xiaoang@csu.edu.cn (A.X.); 2 Light Alloy Research Institute, Central South University, Changsha 410083, China; 3 State Key Laboratory of High Performance Complex Manufacturing, Central South University, Changsha 410083, China

**Keywords:** 7075-T6 aluminum alloy, electromagnetic forming, hot forming, numerical simulation, mechanical property

## Abstract

High-strength 7075 aluminum alloy is widely used in the aerospace industry. The forming performance of 7075 aluminum alloy is poor at room temperature. Therefore, hot forming is mainly adopted. Electromagnetic forming is a high-speed forming technology that can significantly improve the forming limit of difficult-to-deform materials. However, there are few studies on electromagnetic hot forming of 7075-T6 aluminum alloy. In this study, the deformation behavior of 7075-T6 aluminum alloy in the temperature range of 25 °C to 400 °C was investigated. As the temperature increased, the sheet forming height first decreased, then increased. When the forming temperature is between 200 °C and 300 °C, η phase coarsening leads to a decrease in stress and hardness of the material. When the forming temperature is between 300 °C and 400 °C, continuous dynamic recrystallization of 7075 aluminum alloy occurs, resulting in grain refinement and an increase in stress and hardness. The results of numerical simulations and experiments all show that the forming height and deformation uniformity of the sheet metal are optimal at 400 °C, compared to 200 °C.

## 1. Introduction

With rapid developments in aerospace engineering, high-speed trains, and large aircraft, the demand for high-strength, lightweight structural parts is increasing. High-strength and lightweight alloy structural parts can improve material properties. High-strength aluminum alloys offer the advantages of low density and high strength [1,2] and are widely used in the aerospace industry. However, poor plasticity of high-strength aluminum alloys at room temperature limits their application [3,4].

Under hot forming conditions, the yield stress of high-strength aluminum alloys decreases dramatically, and the plastic deformation ability increases. Xiao et al. [5] studied the hot compression test of an Al-Zn-Mg-Cu alloy in the deformation temperature range of 350–450 °C and the strain rate range of 0.001–1 s^−1^. Based on the microstructure characteristics, the optimum processing parameters were obtained in the temperature range of 380–405 °C and the strain rate range of 0.006–0.035 s^−1^. Wang et al. [6] studied the formability of AA2024 aluminum alloy at temperatures ranging from 350 °C to 493 °C using tensile tests. The highest plastic forming performance was observed at 450 °C. However, the ductility of the material sharply declined when the temperature exceeded 450 °C. This is due to precipitation of solute atoms on grain boundaries, resulting in grain boundary separation, which reduces the ductility of the material. Kumar et al. [7] studied the tensile properties of 7020-T6 aluminum alloy in a temperature range of 150 °C to 250 °C. The plastic deformation capacity increased with increasing temperature. However, at temperatures higher than 150 °C, the material exhibited dynamic recovery and η′ phase coarsening, resulting in a decrease in tensile and yield stress. Tomoyoshi et al. [8] studied the influence of temperature on the stamping of 2024 aluminum alloy. When the temperature was 400 °C, springback in the stamping part was reduced to zero. However, when the temperature rose to 500 °C, the sheet fractured as the temperature was close to the solidus temperature. Behrens et al. [9] studied the formability of 7022 and 7075 aluminum alloys at temperatures ranging from 150 °C to 300 °C. At 300 °C, the maximum strain increased. Xiao et al. [10] studied the formability of 7075 through hot uniaxial tensile tests. The forming limit was higher at 400 °C than at 20 °C, 450 °C, and 500 °C. Sławomi et al. [11] studied the formability of 7075-T6 aluminum alloy vehicle supports between 20 °C and 240 °C. The parts broke at 20 °C. When the forming temperature was 240 °C, parts were formed accurately, and the tensile stress of the material was reduced by 11% compared with the original state. Huo et al. [12] studied the formability and the microstructural evolution of 7075-T6 sheets at temperatures ranging from 20 °C to 250 °C through sheet bulging experiments. The results showed fine η′ phase and GP zones at 200 °C, leading to the best formability.

Electromagnetic forming (EMF) is a high-speed forming technology that utilizes pulse magnetic field force to process metal [13,14,15]. In their classic review paper, Psyk et al. [16] pointed out that EMF can greatly reduce springback and improve the forming limit of materials compared with traditional quasi-static forming. Liu et al. [17] formed V-shaped parts with flanges through electromagnetic-assisted stamping (EMAS) using 5052 aluminum alloy. A uniform pressure coil was used to apply pulsed magnetic pressure to the whole part, which effectively reduced tensile stress on the outer surface and compressive stress on the inner surface. Cui et al. [18] proposed EMAS with magnetic force reverse loading. When the die structure did not change, springback in the V-shaped 5052 aluminum alloy part after stamping was reduced. The results show that the equivalent plastic strain and plastic dissipated energy increase after EMF, whereas the stress and elastic strain energy decrease. Cui et al. [19] proposed electromagnetic increment forming (EMIF), which was used to form large 3003 aluminum alloy parts with a moving discharge of small coils. Cui et al. [20] proposed an electromagnetic partitioning forming technology that can precisely manufacture and achieve springback control of large bending radius parts using 3003 aluminum alloy. During the process, elastic deformation is transformed into plastic deformation, and the residual stress is very small after EMF.

A great deal of research has been carried out on the formability of materials. Feng et al. [21] used a V-shaped die and tapered die to perform EMF of 5052 aluminum alloy plates. Compared with quasi-static stamping, maximum strain was increased by about 30% in the side wall of the V-shaped specimens and by about 100% in the tapering specimen. Fang et al. [22] performed drawing experiments using EMIF and 5052 aluminum alloy. Compared with quasi-static stamping, the drawing height was improved by 140% and the dislocation density was increased. Su et al. [23] compared the forming limits of 2219-O aluminum sheets under quasi-static, uniaxial electromagnetic force tensile, and dynamic tensile loads through unidirectional tensile experiments. Under the uniaxial electromagnetic force tensile condition, the forming limit of 2219-O sheets increased by 45.4% compared with the quasi-static tensile condition, and 3.7% to 4.3% compared with the dynamic tensile loading. Electromagnetic hot forming has also been proposed for difficult-to-deform materials. Xu et al. [24] studied the formability of AZ31 magnesium alloy sheets at different temperatures. When the temperature increased from 25 °C to 200 °C, the forming limit increased. However, when the temperature rose to 250 °C, the forming limit decreased. In contrast to 25 °C and 250 °C, grain refinement was clearly observed at 200 °C. Therefore, electromagnetic hot forming is an effective way to improve the formability of difficult-to-deform materials. However, previous research has mainly focused on aluminum alloy materials with good room-temperature plasticity. There are fewer studies on electromagnetic hot forming of high-strength aluminum alloys such as 7075 aluminum alloy.

In this paper, electromagnetic hot forming of 7075-T6 aluminum alloy is proposed. The deformation behavior and mechanical properties of sheet metal were studied at different temperatures both macroscopically and microscopically via experimentation, and the forming process was analyzed using numerical simulations.

## 2. Experiment

### 2.1. Experimental Apparatus

**Figure 1:**
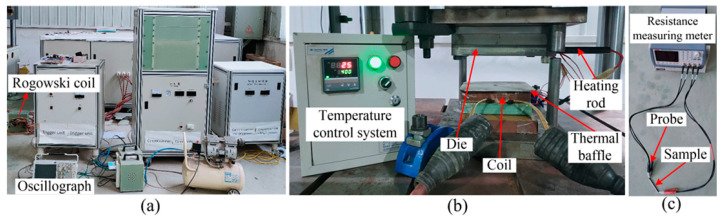
Experimental apparatus: (**a**) electromagnetic forming machine; (**b**) forming apparatus; (**c**) resistance measuring instruments.

Figure 1a shows the 200 kJ Electromagnetic Forming (EMF) machine (Central South University, Changsha, Hunan, China)and 100-ton hydraulic press (Zhongyou, Tengzhou, Shandong, China) used in the experiment. The rated voltage and capacitance of the EMF machine were 25 kV and 640 μF, respectively. A Rogowski coil and oscilloscope were used to measure the discharge current flowing through the coil. Figure 1b shows the experimental tool used for electromagnetic hot forming, including the coil, sheet, and forming die. During the hot forming process, the sheet is first heated in a heating stove, and then the mold-contained heating rods are used to press the sheet. Figure 1c shows the material resistance tester (Anbai, Changzhou, Jiangsu, China). In this study, the forming temperatures were set to 25 °C, 200 °C, 300 °C, and 400 °C. The resistivity of aluminum alloy at different temperatures was obtained using a resistance tester.

**Figure 2:**
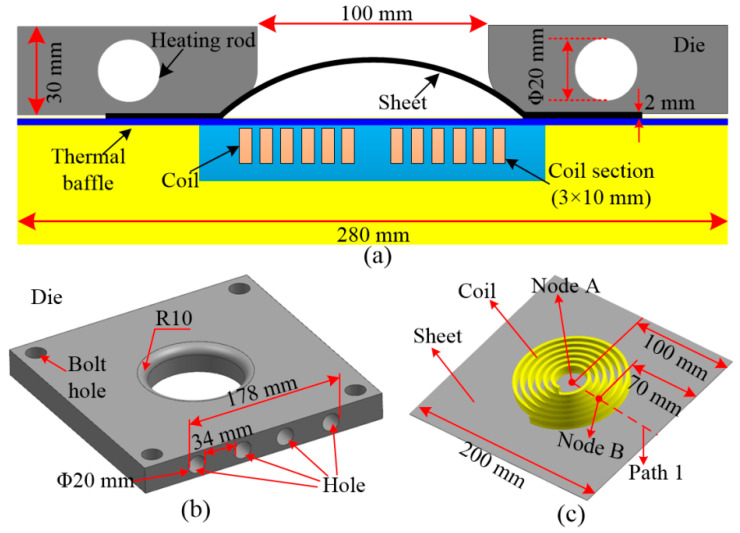
Structure of electromagnetic hot forming: (**a**) forming diagram; (**b**) forming die; (**c**) coil and sheet.

Figure 2a shows the 2D structure of electromagnetic hot forming. The coil is a spiral structure. The cross section (length × width) of the coil wire was 3 mm × 10 mm, and the distance between each turn of the coil was 3 mm. Figure 2a shows a three-dimensional (3D) drawing of the forming die. The diameter of the center opening of the forming die was 100 mm, and the radius of the fillet was 10 mm. Four holes were arranged on the side of the forming die to install the heating rod. The material used in the experiment was 7075-T6 aluminum alloy with the following dimensions: 200 mm × 200 mm × 1 mm. To facilitate subsequent analysis, Path 1 on the sheet was selected. Two special nodes on Path 1 were selected. Node A is located at the sheet center, which is 100 mm away from the sheet edge. Node B is 70 mm away from the sheet edge, as shown in Figure 2c.

**Figure 3:**
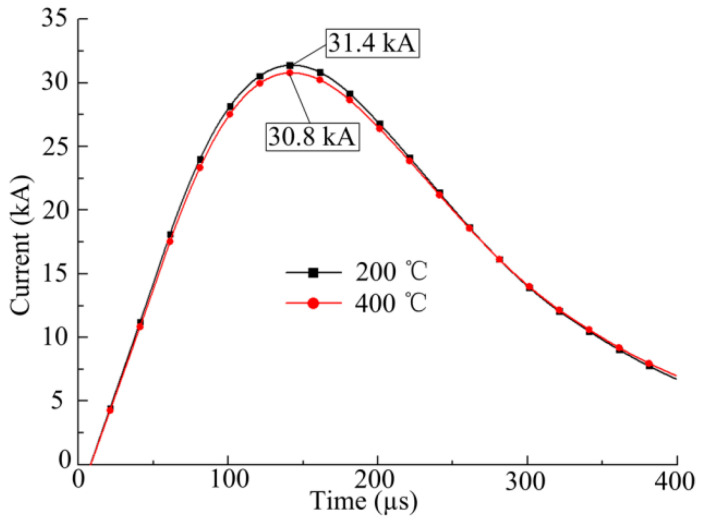
Current curve at 5 KV.

Figure 3 shows the current curves passing through the coil at different temperatures, when the discharge voltage is 5 kV. When the sheet temperature increases, the sheet resistance increases. The mutual inductance between the coil and the sheet decreases, while the total inductance of the discharge system increases with the sheet temperature increase. Thus, the peak current values decrease slightly at 400 °C compared with 200 °C.

### 2.2. Analysis of Forming Results

**Figure 4:**
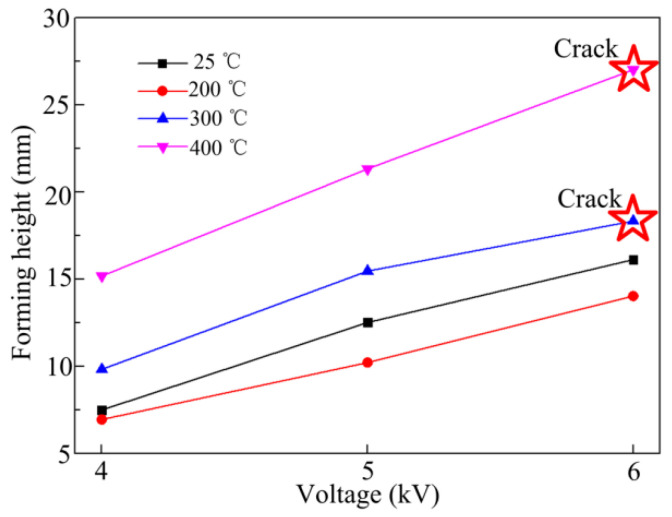
Change in sheet metal deformation with voltage at different forming temperatures.

Figure 4 shows the relationship between maximum forming height of sheet metal and voltage at different temperatures. The forming height increases with increasing voltage. The same trend was observed at each temperature. The forming height of sheet metal first decreased, then increased with increasing temperature. The forming height was lowest at 200 °C and highest at 400 °C.

**Figure 5:**
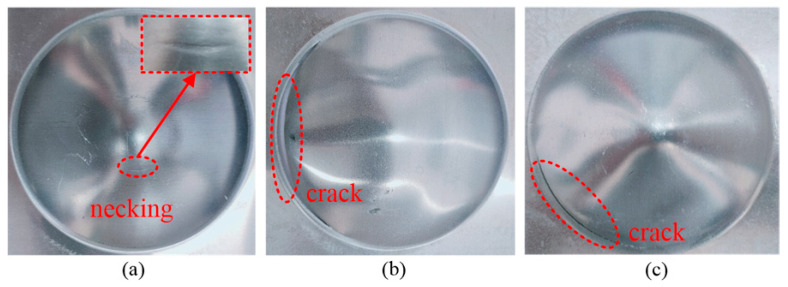
Fracture types at different voltages and temperatures: (**a**) 25 °C, 8 kV; (**b**) 300 °C, 6 kV; (**c**) 400 °C, 6 kV.

Figure 5 shows the fracture types of the sheet metal for different voltages and temperatures. When the forming temperature was 25 °C and the discharge voltage was 8 kV, necking occurred at the top of the sheet metal. When the forming temperature was 300 °C and 400 °C, the edge of the sheet metal cracked under a discharge voltage of 6 kV.

**Figure 6:**
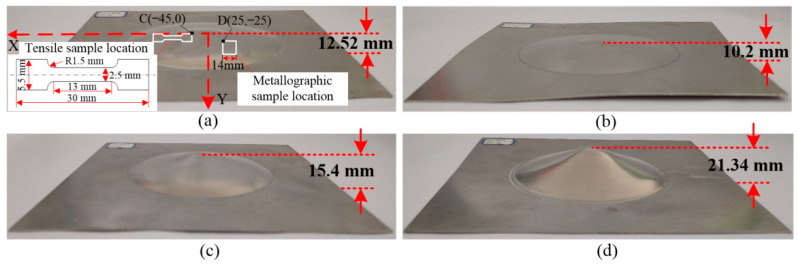
Experimental results for different forming temperatures at a voltage of 5 kV: (**a**) 25 °C; (**b**) 200 °C; (**c**) 300 °C; (**d**) 400 °C.

The forming results of sheet metal at different temperatures with a discharge voltage of 5 kV are shown in Figure 6. The forming heights at 25 °C, 200 °C, 300 °C and 400 °C were 12.5 mm, 10.2 mm, 15.4 mm and 21.3 mm, respectively. As the temperature increased, the forming height of the sheet metal first decreased, then increased. Compared with 25 °C, the forming height of sheet metal decreased by 18.5% at 200 °C, while the forming heights at 300 °C and 400 °C increased by 23% and 70.4%, respectively.

**Figure 7:**
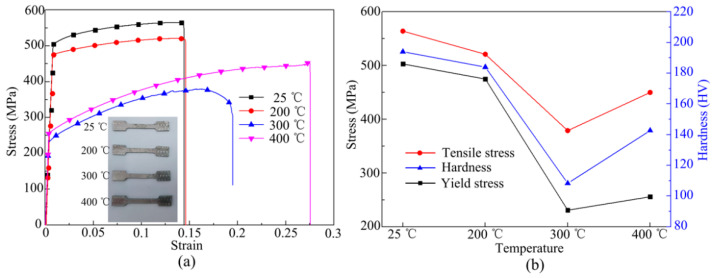
Mechanical properties of sheet metal after forming: (**a**) tensile curve; (**b**) stress and hardness curves.

After forming at 5 kV, mechanical properties of 7075-T6 sheets at different temperatures were examined. The tensile sample was cut with point C (45, 0) as the reference point, and the hardness sample was cut with point D (−25, −25) as the reference point, as shown in Figure 6a. Figure 7 shows the tensile curves and deformed samples obtained with different forming temperatures. At 25 °C, the yield stress, tensile stress, and hardness of the deformed material were 503 MPa, 560 MPa, and 194 HV, respectively. At 200 °C, the values were 475 MPa, 521 MPa, and 184 HV, respectively. At 200 °C, the yield stress, tensile stress, and hardness of the material decreased by 5.5%, 7%, and 5.2%, respectively, compared with 25 °C. At 300 °C, the yield stress, tensile stress, and hardness of the deformed material were 231 MPa, 379 MPa, and 108.3 HV, respectively. At 400 °C, the values were 256 MPa, 450 MPa, and 142.7 HV, respectively. The mechanical properties of the material were lowest when the forming temperature was 300 °C. In addition, the forming height was lower at 200 °C compared with 25 °C. Therefore, the maximum forming height of the sheet metal and good mechanical properties were obtained with a forming temperature of 400 °C.

## 3. Finite Element Simulation

**Figure 8:**
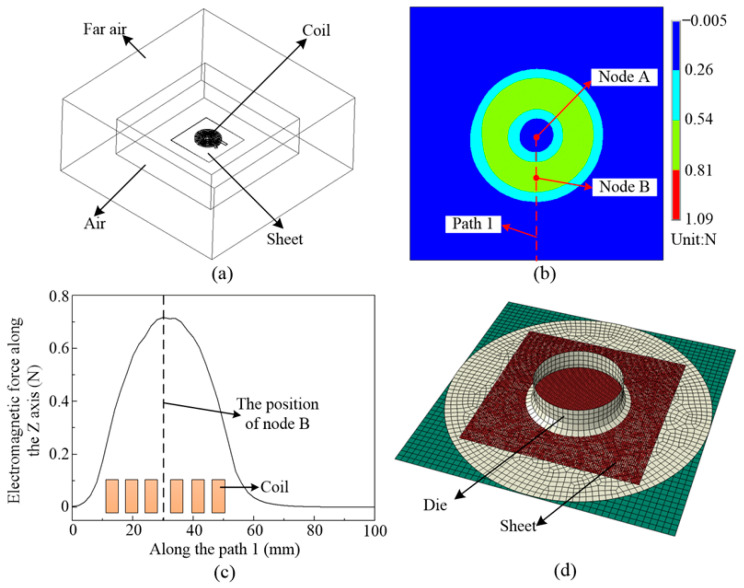
Finite element model of electromagnetic forming: (**a**) electromagnetic field model; (**b**) contours of magnetic force distribution; (**c**) magnetic force along Path 1; (**d**) structure field.

A finite element model for electromagnetic field analysis was established in an ANSYS/EMAG (14.0) module, as shown in Figure 8a. The model includes the coil, sheet material, air field, and far field air. Figure 8b shows the electromagnetic force distribution on the sheet at 400 °C. Figure 8c shows the magnetic force distribution along Path 1. The magnetic force generated at node A is close to 0, while the largest magnetic force occurs at node B. A finite element model of the deformation field was established in ABAQUS/EXCRITE (6.13), as shown in Figure 8d. The model consists of a die, a blank holder, and a sheet. To shorten the computation time, the die and blank holder were set as rigid bodies. In the simulation process, the electromagnetic force calculated in ANSYS (14.0) was imported into ABAQUS (6.13) to analyze the deformation of the sheet material. Then, the deformation results were imported into ANSYS/EMAG (14.0) to calculate the electromagnetic force for the next step.

**Figure 9:**
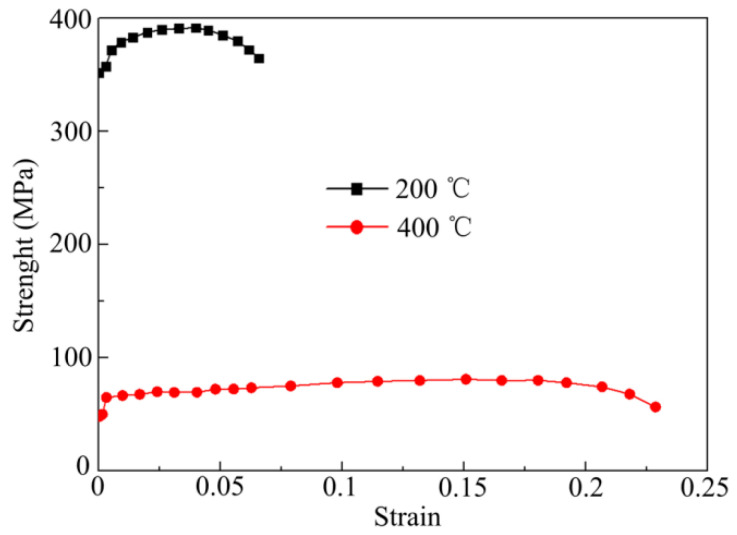
Stress–strain of materials at different temperatures.

The forming results at different temperatures (200 °C and 400 °C) were numerically simulated. The resistivity of the materials at 200 °C and 400 °C is 9.2 × 10^−8^ Ω·m and 12.9 × 10^−8^ Ω·m, respectively. Figure 9 shows the stress–strain curves of 7075-T6 aluminum alloy at different temperatures. To investigate the influence of strain rate on the mechanical properties of the materials, the Johnson–Cook constitutive model was adopted, expressed as

(1) $$\sigma =\sigma_{s}\lfloor 1+C\ln\varepsilon^{*}\rfloor$$

where $\sigma_{s}$ is the stress curve of different temperatures under quasi-static conditions (see Figure 9), and C is the strain rate coefficient. For 7075-T6 aluminum alloy, C = 0.034. $\varepsilon^{*}$ is the strain rate of the material.

**Figure 10:**
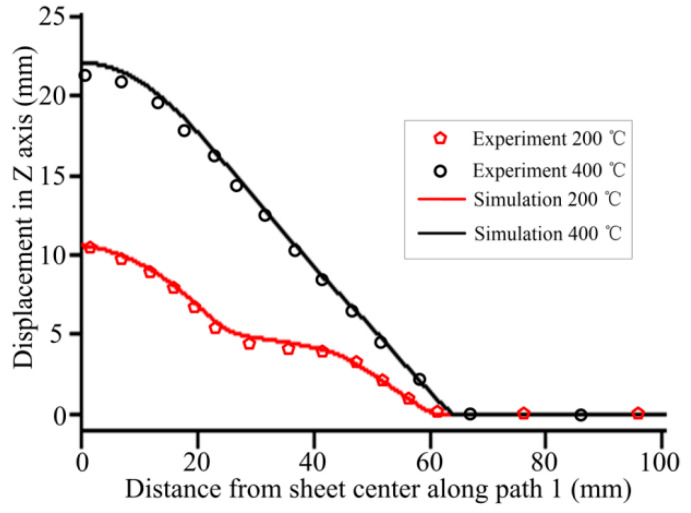
Comparison of simulated and experimental deformed profiles.

Figure 10 shows the sheet deformation profiles obtained by experimentation and numerical simulation. At 200 °C and 400 °C, the experimental forming heights are 10.2 mm and 21.34 mm, respectively. The forming heights by simulation are 10.7 mm and 22.14 mm, respectively. The maximum errors between the simulation and experiment are less than 6% at four temperatures. The simulation results are consistent with the experimental results. Moreover, the deformation profile at 200 °C is wavy, whereas the deformation profile at 400 °C is smooth.

**Figure 11:**
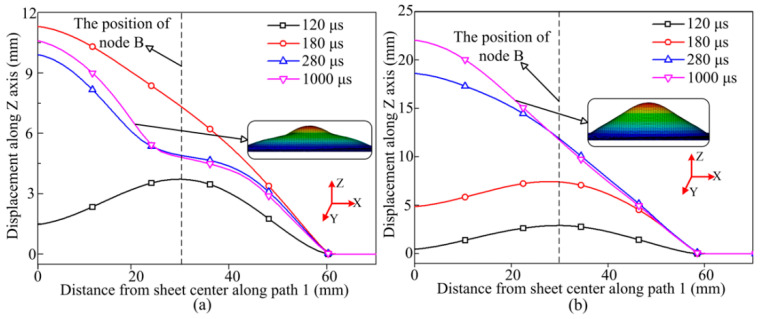
Deformation profile of sheet material at different time points during the forming process at different temperatures: (**a**) 200 °C; (**b**) 400 °C.

From Figure 4, Figure 5, Figure 6,Figure 7 and Figure 10, the forming height of the sheet at 200 °C is the lowest, and the sheet deformation uniformity is poor. At 400 °C, the deformation uniformity and forming height of the parts are the best. Therefore, the deformation process was compared between 200 °C and 400 °C. Figure 11a shows the sheet deformation profile at different time points during the forming process at 200 °C. The sheet forming height is highest at 180 μs, and then vibration appears in the sheet, resulting in a wavy profile. Figure 11b shows the deformed profile at different time points during the forming process at 400 °C. Yield stress in the material is greatly reduced, leading to a significant reduction in the vibration of the material during deformation, compared with 200 °C. At the forming temperature of 400 °C, when the deformation is terminated, the deformation profile is uniform.

**Figure 12:**
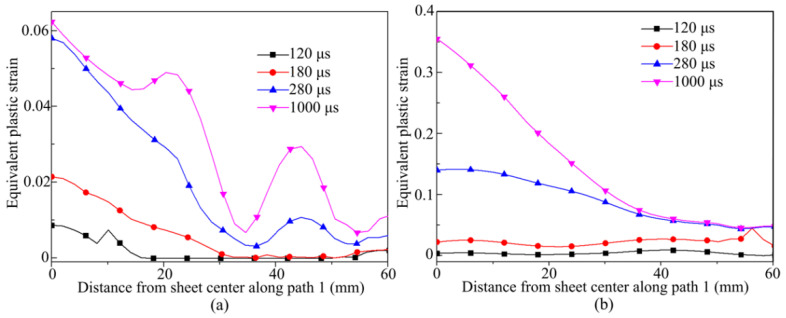
Equivalent plastic strain of sheet material at different time points during the forming process at different temperatures: (**a**) 200 °C; (**b**) 400 °C.

Figure 12a shows the equivalent plastic strain on the sheet at 200 °C at different times. After 180 μs, the equivalent plastic strain on the sheet increases. The equivalent plastic strain appears wavy at 1000 μs. Figure 12b shows the equivalent plastic strain on the sheet at 400 °C at different times. After 180 μs, the equivalent plastic strain increases sharply over time. The strain on the sheet is uniformly distributed. At 1000 μs, the greater the distance away from the sheet center, the smaller the plastic strain appears.

**Figure 13:**
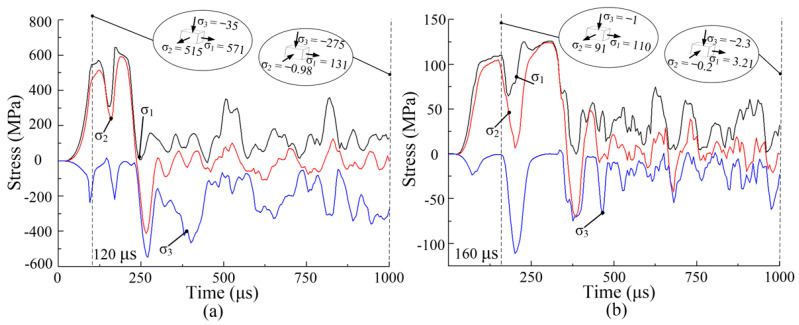
Variation of three principal stresses with time at node A during the forming process at different temperatures: (**a**) 200 °C; (**b**) 400 °C.

Figure 13 shows three principal stresses with time of node A at a voltage of 5 kV and forming temperatures of 200 °C and 400 °C. σ_1_ is along the tangential direction of the sheet, σ_2_ is along the width direction of the sheet, and σ_3_ is along the thickness direction of the sheet. At 200 °C, σ_1_ reaches its first peak of 571 MPa at 120 μs, and σ_2_ and σ_3_ reach 515 MPa and −35MPa, respectively. At 400 °C, σ_1_ reaches its first peak value of 110 MPa at 160 μs, and σ_2_ and σ_3_ are 91 MPa and −1 MPa, respectively. The stress state of node A is approximately bidirectional isotension. After σ_1_ reaches its first peak, the stress oscillates significantly.

**Figure 14:**
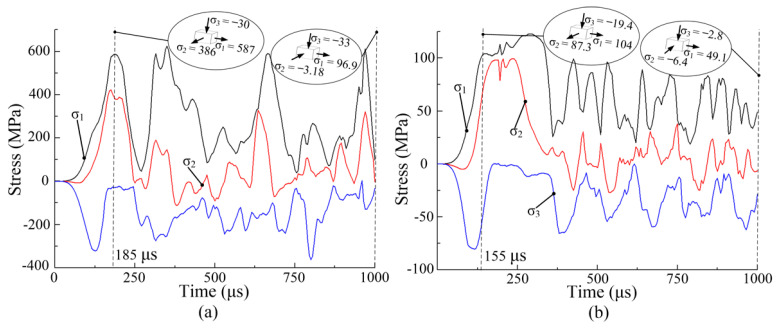
Variation of three principal stresses with time at node B: (**a**) 200 °C; (**b**) 400 °C.

Figure 14 shows three principal stresses and time curve of node B at a voltage of 5 kV and forming temperatures of 200 °C and 400 °C. At 200 °C, σ_1_ reaches its first peak value of 587 MPa at 185 μs, and σ_2_ and σ_3_ are 386 MPa and −30 MPa, respectively. At 400 °C, σ_1_ reaches its first peak value of 104 MPa at 155 μs, and σ_2_ and σ_3_ are 87.3 MPa and −19.4 MPa, respectively. Node B is subjected to bidirectional tensile stress. Similar to node A, the stress oscillates and gradually decreases after the first peak. From Figure 13 and Figure 14, the three principal stresses first increase and then oscillate and decay. Moreover, smaller stresses appear on the sheet after the sheet deformation at 400 °C, compared with 200 °C. Therefore, the sheet forming at a higher temperature can obviously reduce the internal stress of sheet metal. Many scholars have found that stress oscillation leads to a large decrease in the internal stress of the sheet during the EMF process, which can reduce the springback of the sheet [25,26,27].

**Figure 15:**
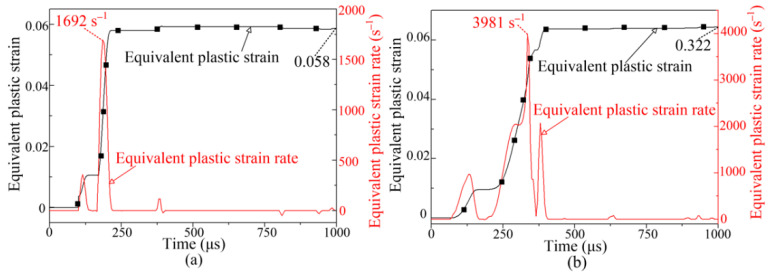
Equivalent plastic strain and strain rate: (**a**) 200 °C, node A; (**b**) 400 °C, node A.

Figure 15a,b show the equivalent plastic strain and plastic strain rate of node A when the discharge voltage is 5 kV. At 200 °C, the maximum equivalent plastic strain and plastic strain rate of node A are 0.058 and 1692 s^−1^, respectively. At 400 °C, the maximum equivalent plastic strain and plastic strain rate of node A are 0.322 and 3981 s^−1^, respectively. The equivalent plastic strain increases at first and then remains stable. Compared with a forming temperature of 200 °C, a larger plastic strain and strain rate in the sheet metal were obtained at 400 °C. This is because yield stress decreases with increasing temperature, leading to greater plastic deformation of the sheet metal at 400 °C.

## 4. Discussion of Results

**Figure 16:**
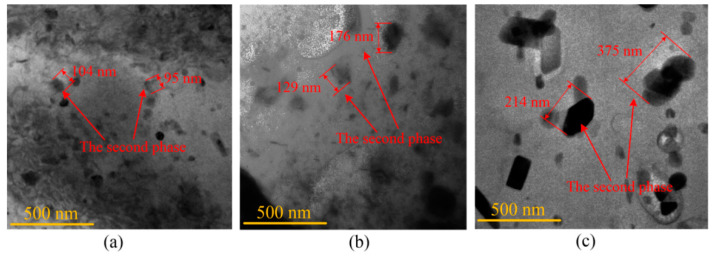
The distribution of the second phase particles: (**a**) 200 °C; (**b**) 300 °C; (**c**) 400 °C.

The microstructure of the deformed sheet was observed and analyzed through a transmission electron microscope (TEM). The TEM samples were prepared by the electrolytic double-spray method. The electrolytic double-spray solution was a mixture of 30% nitric acid and 70% methanol. The temperature was controlled by liquid nitrogen at −35 °C–−25 °C. Figure 16 shows the changes in size of the second phase of the material at 200 °C, 300 °C, and 400 °C. In the forming process of the 7075-T6 aluminum alloy, the main second phase is the η phase. With the increase of temperature, the η phase will be coarser. The increase of η phase size will weaken the strengthening effect and decrease the mechanical properties, as shown in Equation (2) [28]:

(2) $$\sigma =C•f_{t}^{1/2}•r^{-1}$$

where *σ* represents precipitate hardening, *C* is material constants, *f_t_* is the volume fraction of precipitates, and *r* is the particle size.

**Figure 17:**
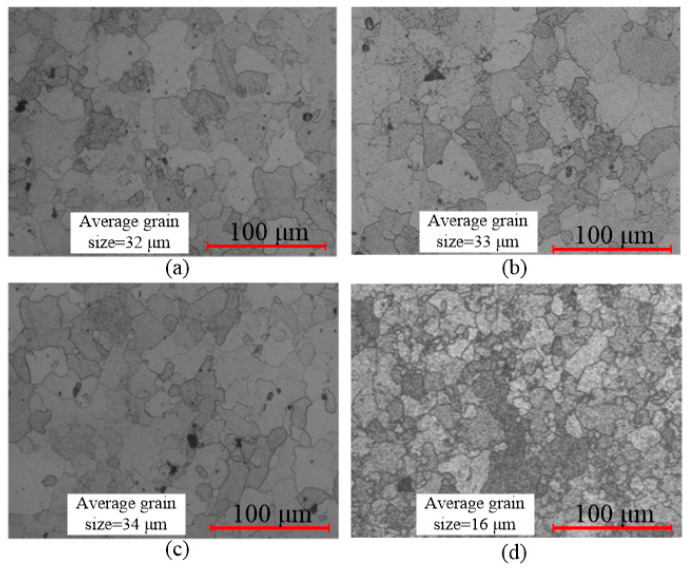
Grain distribution in sheet metal with different forming temperatures: (**a**) 25 °C; (**b**) 200 °C; (**c**) 300 °C; (**d**) 400 °C.

Compared with 25 °C, 200 °C, and 300 °C, the second phase was further coarsened at 400 °C. Thus, the change of the second phase at 400 °C was not the main factor affecting mechanical properties of the material. In order to analyze the reason for the improvement of mechanical properties at 400 °C, the changes of grain size with temperature were analyzed. The metallographic experiments were performed with an optical digital microscope. Figure 17 shows the grain distributions after sheet forming. The average grain size was about 32 μm, 33 μm, 34 μm, and 16 μm at 25 °C, 200 °C, 300 °C, and 400 °C, respectively. The grain refinement occurs at 400 °C compared with other forming temperatures. This is because the dynamic recovery effect appears at 400 °C for 7075 aluminum alloy [29]. Based on the Hall–Petch relationship (Equation (3)) [30], the mechanical properties of the material improve if the grain size decreases. Therefore, the material stress and hardness increase as the forming temperature increases from 300 °C to 400 °C.

(3) $$\sigma =\sigma_{0}+k•d^{-1/2}$$

where σ and $\sigma_{0}$ represent the yield stress and the friction stress when dislocations glide on the slip plane, respectively, *k* represents material constants, and *d* is the grain size.

## 5. Conclusions

7075 aluminum alloy is easy to break at room temperature. In order to increase the forming height of 7075 aluminum alloy, electromagnetic hot forming is proposed. The simulation and experimental results at 25 °C, 200 °C, 300 °C, and 400 °C were compared to prove the accuracy of the FEM model. The main conclusions can be summarized as follows:

(1) Compared with a forming temperature of 200 °C, a more uniform deformation profile and larger plastic strain and strain rate, as well as smaller stresses in the sheet metal, were obtained at 400 °C.
(2) When the temperature was increased from 25 °C to 400 °C, the deformation height first decreased, then increased. The lowest deformation height occurred at 200 °C.
(3) When the forming temperature was increased from 25 °C to 400 °C, the stress and hardness first decreased, then increased. The least favorable mechanical properties occurred at 300 °C. This is because of obvious coarsening in the second phase at 300 °C. Continuous dynamic recrystallization of 7075 aluminum alloy occurs at 400 °C, which leads to grain refinement and improved mechanical properties.

## Data Availability

Data is contained within the article.
